# “Ask” or “Inquire”: operationalizing speech formality in psychosis and its risk states using etymology

**DOI:** 10.1038/s44277-024-00018-5

**Published:** 2024-10-18

**Authors:** Matthew Cotter, Alessia McGowan, Zarina Bilgrami, Cansu Sarac, Johanna Bayer, Jessica Spark, Marija Krcmar, Melanie Formica, Kate Gwyther, Jessica Hartmann, Sophia Shuster, Alexandria Selloni, Jai Shah, Shaynna N. Herrera, Patrick McGorry, Alison R. Yung, Barnaby Nelson, Romina Mizrahi, Guillermo Cecchi, Stephen Heisig, Agrima Srivastava, Cheryl M. Corcoran

**Affiliations:** 1 Department of Psychiatry, Icahn School of Medicine at Mount Sinai, New York, NY USA; 2 Department of Psychology, Emory University, Atlanta, GA USA; 3 School of Health Professions, Long Island University, New York, NY USA; 4 Donders Institute for Brain, Cognition and Behavior, Radboud University, Nijmegen, Netherlands; 5 Orygen, The National Centre of Excellence in Youth Mental Health, University of Melbourne, Parkville, VIC Australia; 6 Centre for Youth Mental Health, University of Melbourne, Parkville, VIC Australia; 7 Department of Public Mental Health, Medical Faculty Mannheim, Central Institute of Mental Health, Heidelberg University, Heidelberg, Germany; 8 Department of Psychiatry, McGill University, Montreal, QC Canada; 9 School of Medicine, Deakin University, Waurn Ponds, VIC Australia; 10 Computational Psychiatry and Neuroimaging, IBM, Yorktown Heights, NY USA

**Keywords:** Human behaviour, Data processing

## Abstract

Many individuals with psychotic symptoms have less complex language than healthy individuals. Word etymology is a lexical feature that has not yet been studied in clinical populations, but among healthy individuals, words of Old French origin are chosen over Germanic-origin words to convey formality (e.g. “inquire” vs. “ask”). Differences in language complexity among individuals with psychotic symptoms may relate to differences in etymological content in speech. Here, we determined the proportion of Germanic-origin word use and Old-French-origin word use in a large cohort of individuals with recent-onset psychosis or at clinically high risk for psychosis, hypothesizing that individuals with recent onset psychosis would have increased use of Germanic-origin words and decreased use of Old-French-origin words. This hypothesis was borne out, even after adjusting for sex, age, recruitment site, education, racial identity, and for a subset, IQ. Etymology proportions were associated with role but not social functioning in individuals with psychotic symptoms, consistent with the premise that they reflect speech formality. Understanding speech differences in the psychosis spectrum through the lens of etymology may lead to new interventions to improve role functioning.

## Lay Summary

When people converse, there is a tendency to use more words of Old French origin (“inquire”, “intelligent”) as opposed to words of Proto-Germanic origin (“ask”, “smart”) when the conversation requires formality. In analyzing the speech etymology content of people experiencing psychotic symptoms, we found their speech to be less formal than healthy controls. We also found an association between less formal speech and reduced role functioning, implying that speech differences could impact academic and/or career success.

## Introduction

Disordered communication is a hallmark characteristic of psychosis, one that is associated with functional deficits [1–3]. One reason for this may be differences in the perceived formality of speech. Speech etymology may approximate formality: in spoken language, healthy individuals tend to choose words of Latinate origin when they feel obliged to speak more formally, e.g. Latinate “intelligent” vs. Germanic “smart” [4]. People are likely to expect that the use of more Latinate words signals a more formal conversation, such as with an employer or stranger [5]. Etymology has also been examined in classic novels, as authors have used different proportions of Germanic- vs. Latinate-origin English words to convey differences in the perceived formality or intellect of characters’ speech [6, 7].

Here we present, to our knowledge, the first analysis of speech etymology content in a clinical cohort. We created a pipeline for pre-processing and analyzing a body of text for its etymology content and compared the results of two methods for determining etymology. This pipeline was applied to transcripts of open-ended interviews conducted with a large, international cohort of English-speaking individuals with psychotic symptoms who either have recent onset psychosis (ROP) or are at clinically high risk for psychosis (CHR), and healthy individuals (HC) similar in demographics for comparison. Many Latinate-origin words owe their association of formality to their use by the English aristocracy after the Norman French invasion of 1066, entering the English lexicon via Old French [4]. We, therefore, compared proportions of Germanic-origin word use to Old-French-origin word use, intending to capture the use of Latinate words that have a heightened association with formality. As Germanic-origin words are more common than Old-French-origin words in English, we also tested whether different patterns of etymology content are related to lexical diversity, measured with Honoré’s Statistic [8]. Honoré’s Statistic measures vocabulary breadth, normalized by text length, and has been associated with clinical measures of poverty of speech in individuals with schizophrenia [9]. Further, as words’ etymology may be associated with their rarity, we also measured perplexity, which at the level of the whole transcript, is indexed by the rarity of each word used by the participant, normalized to account for the fact that English has a few very common words (e.g. “is”, “go”) and many rare ones (e.g. “synecdoche”, “epithalamion”).

We hypothesized that speech from individuals with ROP would contain proportionately more Germanic words and fewer Old French words, and would exhibit less lexical diversity and lower perplexity (less rarity) than HC speech, on the basis that those with ROP would have greater difficulty handling the cognitive load of varying one’s speech and retrieving uncommon words. We also predicted that greater use of Germanic words and less use of Old French words would be associated with less lexical diversity and less perplexity. While there were no specific hypotheses regarding the CHR cohort, it was included in analyses as findings may be informative about the pathophysiology of schizophrenia in a developmental context. We tested for associations with sex, age, recruitment site, education duration, racial identity, antipsychotic use, socioeconomic status (using maternal education as a proxy [10]), and IQ in the sub-cohort for which scores were available, adjusting for these covariates when associations were found.

## Materials and Methods

### Study design and setting

This analysis draws from two studies of language production spanning the schizophrenia spectrum. Participants were individuals with ROP (symptom onset within 5 years), youth at CHR, and HC individuals with similar demographics. Data in the single-site study (R01MH107558) were collected between 2016 and 2023 in New York, USA. In the multisite study (R01MH115332), data were collected between 2018 and 2022 at clinical research programs in New York, USA; Melbourne, Australia; and Toronto, Canada. In both studies, open-ended interviews were collected for computational analysis. All interviews were conducted using the same protocol, using qualitative research methods as described previously, and each lasted approximately 30 min [11–13]. Interviewers first asked participants “How have things been going for you lately?” and asked open-ended follow-up questions to encourage further discussion. Studies were approved by the Institutional Review Boards of the Icahn School of Medicine at Mount Sinai and the New York State Psychiatric Institute at Columbia University, as well as at Orygen, The National Centre of Excellence in Youth Mental Health at the University of Melbourne; the Centre for Addiction and Mental Health in Toronto; and now approved under Clinical and Translational Sciences (CaTS) BioBank by the Research Ethics Board of the Centre intégré universitaire de santé et de services sociaux (CIUSSS) de l’Ouest-de-l'Île-de-Montréal – Mental Health and Neuroscience subcommittee. All participants (and their parents or guardians if minors) provided written informed consent.

### Participants

Across the studies, language samples were collected from 92 individuals with ROP, 144 individuals at CHR, and 173 HC. Exclusion criteria included risk of harm to self or others incompatible with research participation, medical or neurological disorders that might affect language, IQ under 70, and for HC individuals only, a DSM Axis I diagnosis. All sites used the Structured Clinical Interview for DSM disorders [14] to determine diagnoses. CHR status was determined using the Structured Interview for Psychosis-Risk Syndromes (SIPS) [15] in North America or the Comprehensive Assessment of At-Risk Mental States (CAARMS) [16] in Australia. We chose to study individuals with ROP to minimize confounding from chronicity and antipsychotic exposure.

### Assessments

Self-reported age, sex, and racial identity were collected. Medication use, including antipsychotic use, was recorded as yes/no. Symptom severity was assessed in individuals with ROP using the Positive and Negative Syndrome Scale (PANSS) [17], and in those at CHR the SIPS was used in New York/Toronto and the CAARMS in Melbourne. HC participants in R01MH107558, in New York, were administered the SIPS. HC participants in R01MH115332 were administered the CAARMS in Melbourne, the SIPS in Toronto, and the PANSS in New York. Functioning was assessed with the Global Functioning: Role and Social scales (GF-R and GF-S), which were developed for use in individuals at CHR [18]. In R01MH115332, IQ was estimated using the Wechsler Abbreviated Scale of Intelligence (WASI) vocabulary and matrix reasoning sub-sections [19].

### Analysis of etymology content

**Fig. 1:**
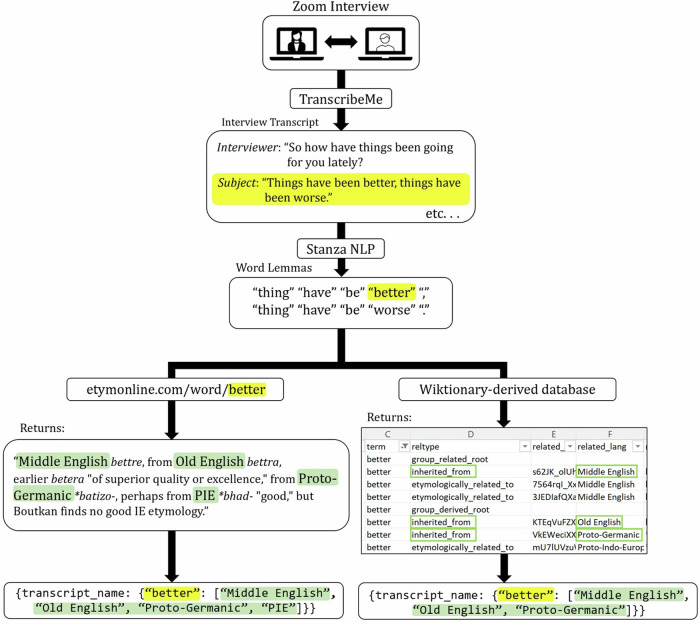
Etymology analysis pipeline. The pipeline of language data collection, preprocessing, and analysis for etymology content is outlined here. Participants engaged in a recorded Zoom interview, which was then transcribed by the HIPAA-compliant transcription service TranscribeMe!. From there, participant speech was isolated, converted to lower case, and lemmatized using the Stanza NLP package for Python. To calculate etymology proportions, word lemmas were searched on Etymonline.com, and the names of origin languages were pulled from the etymology description on the returned webpage. Separately, word lemmas were searched in a database derived from Wiktionary.com, and word origins with the relation types “inherited from”, “derived from”, etc. were retrieved.

Recorded interviews were transcribed by the HIPAA-compliant transcription service TranscribeMe! (www.transcribeme.com; Fig. 1). Audio transcripts were uncapitalized, then lemmatized using the NLP package Stanza [20]. Lemmatization converts different word inflections to their root inflection (e.g., “is” and “am” both become “be”). Special characters and punctuation were removed. Since the etymology of many words is contested, we determined etymologies using two resources: Etymonline.com [21], which is based on a curated set of etymological dictionaries; and a database derived from Wiktionary.com [22], which is open-source and has many contributors.

We determined for each lemma whether its etymology contained a Germanic or Old French language origin. Because determiners (e.g., “which”, “that’) and other common structural parts of speech (“the”, “she”, “it”) are predominantly Germanic in origin [23], analysis of Germanic vs. Old French content of the speech was restricted to nouns, adjectives, verbs, and adverbs, where there are more Germanic and Old French synonyms (e.g. “ask” vs. “inquire”). The quantity of Germanic and Old French word use was calculated as the proportion of these parts of speech that have Germanic or Old French origin in their etymology. Some words had both Germanic and Old French origin, e.g. due to prefixes or suffixes (“talkative”), or neither Germanic or Old French origin (“karaoke”). Only words of exclusively Germanic or Old French origin were included in these calculations.

### Calculation of lexical diversity

Lexical diversity was determined for each preprocessed and lemmatized language sample using Honoré’s Statistic [8]. This scale captures variance in vocabulary normalized by transcript length. The formula for Honoré’s Statistic is:

$$R=\frac{100\log \left(N\right)}{1-{V}_{1}/V}$$

where N is the total text length, V_1_ is the number of words that appear exactly once, and V is the number of unique words. Larger values of R indicate greater lexical diversity.

In keeping with past reports on this calculation [24–26], Honoré’s Statistic was calculated from the whole text of transcripts and was not filtered by part of speech like the etymology proportion and perplexity calculations were.

### Calculation of Perplexity (Rarity)

Within each transcript, all word lemmas assessed for etymology were also assessed for their rarity as a measure of first-order perplexity. First-order perplexity assumes that the probability of a string of words is simply the product of each individual word’s unary probability in a language. This model was chosen to match the context-independent nature of the etymology content analysis. To quantify perplexity, we identified each lemma’s frequency in the Google N-grams database, which contains over one trillion words of text derived from publicly accessible webpages [27]. We derived a perplexity score for each transcript according to the following formula:

$$\log \left(\Pi \right)=\frac{1}{n}{\sum}_{i=1}^{n}\log ({W}_{i})$$

where Π is the perplexity of the entire transcript, n is the number of lemmas (counting only nouns, verbs, adjectives, and adverbs), and w_*i*_ is a given word’s probability based on its frequency in the Google N-grams database [28].

### Statistical analyses

#### Assessing and adjusting for potential covariates

We tested sequentially for associations of lexical variables with sex, age, recruitment site, education, racial identity, socioeconomic status, and IQ (in the subset where available), and where we found associations with etymology content, we adjusted all lexical variables for association with that covariate. Socioeconomic status was approximated using maternal education, a common proxy [10]. For categorical variables (sex, recruitment site, racial identity), we tested for associations using t tests or ANOVAs and adjusted lexical variables by the difference of HC median scores in each category. Because the cohorts of individuals identifying as Black and “Other/more than one race” were small and did not differ from one another on any lexical variable, these cohorts were combined for adjustment. For continuous variables (age, education, socioeconomic status, IQ), we tested for associations using Spearman correlations and adjusted scores using residuals from linear regression models trained on HC data.

Antipsychotic use (dichotomized as yes/no) was assessed as a covariate in the CHR and ROP cohorts separately. No HC used antipsychotic medications.

#### Group differences

After adjustments for covariates, ANOVA was used to test for significant differences between HC, CHR, and ROP cohorts in etymology proportion, as well as for lexical diversity (Honoré’s Statistic) and perplexity. To identify cohorts driving significant group differences, we calculated pairwise independent t tests. Tests were repeated within recruitment sites for significant whole-dataset group differences. We hypothesized differences between HC and ROP across linguistic variables.

#### Correlates and predictors of etymology

We calculated a Pearson correlation matrix between etymology proportions, lexical diversity, and perplexity to determine the proportion of variance in etymology content explained by perplexity and lexical diversity.

To determine the relative contributions of diagnosis, lexical diversity, perplexity, age, sex, recruitment site, education, and racial identity to etymology content, we generated two multiple linear regression models, one with Germanic origin word frequency as the dependent variable, the other with Old French, and compared standardized regression coefficients. These variables were chosen because significant correlations or group differences were found in the analysis of covariates, excepting IQ since it was only available in a subset. These analyses were performed using Jamovi [29].

#### Clinical relevance of etymology patterns

To determine whether lexical features were related to symptom severity in the clinical cohorts, we calculated Spearman’s correlations between etymology proportions and total positive/total negative symptom scores from the SIPS (New York/Toronto) and CAARMS (Melbourne) for the CHR cohort and the PANSS for the ROP cohort. We determined associations of etymology proportions with concurrent functioning by calculating Spearman’s correlations between etymology proportions and GF-R and GF-S scores in the combined CHR and ROP cohort. When correlations were significant, we tested also for associations with lexical diversity and perplexity. We additionally re-tested significant correlations within recruitment sites.

A Bonferroni-corrected significance level of alpha <0.0025 was chosen based on the twenty tests that include multiple linear regression models and correlations with clinical measures.

## Results

### Comparing Etymonline-derived and Wiktionary-derived etymology proportions

As Etymonline.com sources etymologies from curated etymological dictionaries, whereas Wiktionary.com collects open-source contributions, we compared calculated etymology content derived from each. Across all transcripts in our analytic sample, Etymonline-derived and Wiktionary-derived etymology proportions were nearly identical (Germanic: *r* = 0.99, *p* < 0.05; Old French: *r* = 0.99, *p* < 0.05). Since Wiktionary could potentially be used for analyses of etymology in other languages, we opted to use only Wiktionary-derived etymology data for the following analyses.

### Cohort demographics

Participants across the cohorts were largely in their early twenties, and cohorts were approximately equal in their proportion of males and females (Table 1). Mean age differed significantly between all three cohorts (HC/CHR: t(315) = 4.5, p < 0.001; HC/ROP: t(263) = 1.7, p < 0.001; CHR/ROP: t(234) = −2.08; p < 0.001). The HC cohort had a trend for a larger proportion of female participants (X^2^_2_ = 5.53, *p* = 0.06).

**Table 1:** Cohort demographics.

Clinical Cohort	HC	CHR	ROP
Cohort N	173	144	92
Age, average (SD)***	23.4 (4.8)	21.1 (4.6)	22.4 (4.8)
Sex - % Female	62.4	50.0	52.2
% Antipsychotic Rx***	0	15.3	76.1
% Schizophrenia Dx	–	–	68.5
**Years of Education**			
N with education data	166	141	88
Average (SD)***	14.3 (2.7)	12.2 (2.8)	11.9 (2.7)
Racial Identity***			
N with race (and education) data	164	134	86
% Asian	48.2	22.4	15.1
% Black	8.5	8.2	12.8
% White	41.5	56.7	68.6
% Other/More than one race	1.8	12.7	3.5
**Maternal Years of Education**			
N with personal and maternal education (and race) data	159	126	77
Mean (SD)***	14.8 (2.7)	14.0 (2.8)	13.1 (3.2)
**IQ (WASI FSIQ-2)**			
N with education (and race) and IQ data	127	82	57
Mean (SD)***	112.3 (11.6)	106.1 (9.7)	101.5 (11.4)
**SIPS/SOPS**			
N with SIPS/SOPS scores	87	74	14
Positive Symptoms average (SD)***	0.7 (1.2)	12.7 (3.6)	15.1 (3.1)
Negative Symptoms average (SD)***	0.6 (1.3)	11.7 (6.0)	12.7 (4.3)
**CAARMS**			
N with CAARMS scores	74	70	61
Positive Symptoms average (SD)***	1.3 (2.2)	13.0 (3.5)	15.7 (5.5)
Negative Symptoms average (SD)***	1.7 (1.8)	9.4 (2.6)	9.7 (3.8)
**PANSS**			
N with PANSS scores	80	70	91
Positive Symptoms average (SD)***	7.5 (1.2)	13.1 (2.9)	14.9 (5.5)
Negative Symptoms average (SD)***	7.5 (0.9)	12.1 (4.9)	13.6 (6.1)
**Global Functioning Scale**			
N with GFS scores	161	142	91
Role Functioning average (SD)***	8.3 (0.9)	6.5 (1.6)	5.8 (1.9)
Social Functioning average (SD)***	8.2 (0.7)	6.4 (1.4)	6.0 (1.4)

***indicates p < 0.001.

### Group differences after correction

At the whole dataset level, we found associations of Germanic and Old French word use with sex, age, recruitment site, education, and race (all *p* < 0.05), but not antipsychotic use or maternal education (a proxy for socioeconomic status [10]). We adjusted all lexical variables for associations with each covariate except maternal education and antipsychotic use. In the subset where IQ was available, we also found correlations with etymology proportions (both *p* < 0.05), and likewise adjusted. See the Supplemental Materials and Methods for further details.

After adjusting for covariates, HC exhibited less Germanic and more Old French word use than individuals with ROP, as hypothesized (Table 2). This difference extended to CHR individuals, who had similar etymology patterns to individuals with ROP. Also as expected, HC had greater lexical diversity than either of the clinical groups, who again did not differ. Perplexity (e.g., rarity) did not vary across the three groups. This pattern of findings held in the sub-cohort for whom IQ was known and adjusted for. Within recruitment sites, findings were largely similar, except that among New York participants, Germanic word use differences were at trend-level significance. Significant group differences in lexical diversity were identified only among Melbourne participants, with the ROP cohort demonstrating less lexical diversity than HC (Supplemental Materials and Methods).

**Table 2:** Group differences in lexical variables after controlling for sex, age, recruitment site, education, race, and IQ.

Full Cohort	Cohort			Group Differences	Pairwise Differences		
	HC	CHR	ROP	ANOVA F (df=2, 382)	HC/CHR t (df=296)	HC/ROP t (df=248)	CHR/ROP t (df=220)
Cohort N	164	134	86	–	–	–	–
Proportion Germanic Words, mean (SD)	0.701 (0.036)	0.724 (0.031)	0.724 (0.038)	19.3***	−5.4***	−5.8***	−1.2
Proportion Old French Words, mean (SD)	0.190 (0.028)	0.172 (0.025)	0.173 (0.030)	18.8***	4.4***	5.5***	1.6
Honoré's Statistic, mean (SD)	682 (53)	667 (53)	666 (57)	3.7*	1.5	2.4*	1.0
Perplexity, mean (SD)	112 (20)	108 (14)	112 (19)	1.7	–	–	–

Sub-cohort where IQ scores were available	Cohort			Group Differences	Pairwise Differences		
	HC	CHR	ROP	ANOVA F (df=2, 267)	HC/CHR t (df=210)	HC/ROP t (df=184)	CHR/ROP t (df=140)
Cohort N	127	82	57	–	–	–	–
Proportion Germanic Words, mean (SD)	0.700 (0.034)	0.724 (0.030)	0.731 (0.032)	24.0***	−5.4***	−5.8***	−1.2
Proportion Old French Words, mean (SD)	0.190 (0.028)	0.174 (0.025)	0.168 (0.025)	19.5***	4.4***	5.5***	1.6
Honoré's Statistic, mean (SD)	682 (54)	671 (53)	661 (59)	3.3*	1.5	2.4*	1.0
Perplexity, mean (SD)	113 (21)	109 (14)	109 (20)	1.4	–	–	–

Bold = significant difference, with *indicating *p* < 0.05 and ***indicating *p* < 0.001.

### Relationship of etymology content, perplexity, and lexical diversity

Testing for associations among lexical features, we generated a Pearson correlation matrix between etymology proportions, lexical diversity, and perplexity, after adjustment for covariates (Table 3). All features were significantly intercorrelated (p’s < 0.05). Germanic word use and Old French word use were highly negatively correlated. Germanic word use had moderate negative correlations with lexical diversity and perplexity, while Old French word use positively correlated with lexical diversity and perplexity. Lexical diversity and perplexity were themselves weakly positively correlated.

**Table 3:** Pearson correlation matrix of lexical variables.

	Germanic word use	Old French word use	Lexical diversity
Old French word use	−0.88		
Lexical diversity	−0.34	0.33	
Perplexity	−0.37	0.24	0.20

### Multivariate model and proportion of variance attributable to each variable

To determine the relative contributions of clinical cohort, sociodemographic features, lexical diversity, and perplexity to variance in uncorrected etymology proportions, we performed multiple linear regressions and compared standardized coefficient estimates.

Each model captured a moderate portion of the variance in its respective etymology proportion (adjusted R^2^ = 0.39 for Germanic; adjusted R^2^ = 0.33 for Old French; Table 4). Clinical cohort, lexical diversity, and perplexity all contributed significantly to both Germanic and Old French models (all corrected *p* < 0.05), while sex, age, education, and race did not. The recruitment site correlated significantly with Old French word use but not Germanic. Education significantly correlated with the proportion of Germanic word use, but not Old French. In both models, the most significant contributor to etymology content, according to standardized coefficient estimates, was the clinical cohort (ROP vs. HC).

**Table 4:** Multiple linear regression models for etymology proportions.

Proportion Old French Word Use - R = 0.587, R^2^ = 0.345, adjusted R^2^ = 0.326				
Predictor	Estimate	Standard Error	t	Standardized Estimate
Cohort - CHR or HC	−1.02E-2	3.33E-3	−3.0	−0.33*
Cohort - ROP or HC	−1.15E-2	3.69E-3	−3.1	−0.38*
Honoré's Statistic	1.39E-4	2.57E-5	5.4	0.25***
Perplexity	3.22E-4	7.50E-5	4.3	0.19***
Education (years)	1.43E-3	6.80E-4	2.1	0.14
Age	4.42E-5	3.38E-5	1.3	0.08
Sex - Female or Male	−7.59E-3	2.64E-3	−2.9	−0.25
Site – New York or Melbourne	8.16E-3	4.06E-3	2.0	−0.27
Site – Toronto or Melbourne	2.55E-3	3.86E-3	0.7	−0.08
Racial Identity – Black/Other or Asian	−1.11E-2	4.53E-3	−2.5	−0.36
Racial Identity – White or Asian	−6.87E-3	3.18E-3	−2.2	−0.22
Intercept	2.73E-2	1.85E-2	1.5	—

Proportion of Germanic Word Use *-* R = 0.637, R^2^ = 0.405, adjusted R^2^ = 0.388				
Cohort - CHR or HC	1.46E-2	4.11E-3	3.5	0.37**
Cohort - ROP or HC	1.60E-2	4.56E-3	3.5	0.40*
Honoré's Statistic	−1.73E-4	3.17E-5	−5.5	−0.24***
Perplexity	−6.56E-4	9.25E-5	−7.1	−0.30***
Education (years)	−1.65E-3	8.39E-4	−2.0	−0.12
Age	−3.02E-5	4.17E-5	−0.7	0.04
Sex - Female or Male	7.12E-3	3.26E-3	2.2	0.18
Site - New York or Melbourne	−1.56E-2	5.01E-3	−3.1	−0.39*
Site - Toronto or Melbourne	6.76E-3	4.76E-3	−1.4	0.17
Racial Identity – Black/Other or Asian	1.41E-2	5.59E-3	2.5	0.35
Racial Identity – White or Asian	6.99E-3	3.92E-3	1.8	0.18
Intercept	9.23E-1	2.28E-2	40.5	—

Bold = significant difference, with *indicating *p* < 0.05, **indicating *p* < 0.01, and ***indicating *p* < 0.001 after Bonferroni correction.

### Clinical relevance

Within tests of associations of etymology content (adjusted for covariates) with symptoms, Old French word use had a negative association with negative (but not positive) symptoms, albeit only in ROP (but not CHR) individuals, and the association did not survive Bonferroni correction. Similarly, Germanic word use was not associated with positive or negative symptoms in either clinical cohort. By contrast, global role functioning specifically (but not social functioning) was associated in the combined clinical group with greater formality, indexed by both increased Old French word use (rho = 0.26, *p* < 0.01) and decreased Germanic word use (rho = −0.19, *p* < 0.05), associations that clearly survived Bonferroni correction (Table S2). Of note, global role functioning was not associated with lexical diversity or perplexity, suggesting a specific association with formality. Analyses within recruitment sites yielded similar results (see Supplemental Materials and Methods).

## Discussion

In this study, we leveraged NLP techniques and electronic etymology databases to identify differences in the proportions of Germanic and Old French word use in individuals with psychotic symptoms, including those with recent-onset psychosis (ROP) and those at clinical high risk (CHR) for psychosis, as compared with healthy comparison subjects (HC), similar in demographics and ascertained from the same source population. As hypothesized, we found that, in open-ended interviews, ROP individuals use proportionately more Germanic and fewer Old French words on average than do HC. Further, this pattern extended to CHR individuals. These findings held true after adjusting for associations with sex, age, recruitment site, education, racial identity, and IQ. Personal education was related to etymology patterns, but maternal education (a common proxy for socioeconomic status [10]) was not, consistent with vocabulary exposure in school being most relevant to language patterns. While patterns of etymology content were associated with lexical diversity and perplexity, these other linguistic features did not fully account for the decreased formality of spoken language among the clinical cohort, as indexed by increased Germanic-origin word use and decreased Old-French-origin word use. Further, this decreased formality was associated specifically with worse role functioning (but not social functioning), an association unique to this linguistic feature.

It is interesting to consider the extent to which these differences in speech formality may be explained by differences in the complexity of speech, measured here by lexical diversity and first-order perplexity. Certainly, the clinical groups had not only decreased formality but also decreased lexical diversity (though not perplexity). And while the two indices of formality – the use of Germanic and Old French words – were highly correlated (r = 0.88), as expected, each nonetheless had moderate effect size associations with lexical diversity and perplexity (|rho| between 0.24 and 0.37). While lexical diversity and perplexity did not explain group differences in the use of Germanic and Old French words, these linguistic variables had comparable contributions to their variance as did group membership in multivariate models. Elevated Germanic word use (and decreased Old French use) may reflect greater difficulty handling the cognitive load of varying one’s speech (as captured by lexical diversity) and/or of retrieving uncommon words (as captured by perplexity). Individuals at CHR and with ROP may respond to this difficulty by varying their speech less and using more common words that are readily accessed.

While we tested for many variables we thought might be related to etymological patterns of word usage in spoken language across a spectrum of psychosis pathology, our multiple linear regression models nonetheless explained only 33% and 39% of the variance in Old French and Germanic word use, respectively. Additional variance might therefore be explained by participants’ ability to convey formality. Previous studies have indicated an association of Latinate words with heightened formality [4, 5], but our results appear to demonstrate the utility of examining specifically Old French-origin words for assessing formality. Analyses of classical literature [6] have indicated that writers tend to use more words of Old French origin to indicate formality, whereas Germanic-origin words impart informality and sincerity. Writers are often advised to favor words of Anglo-Saxon origin for improved clarity [30]. Our analyses within recruitment sites indicate that these patterns persist in American, Canadian, and Australian linguistic traditions. Regarding differences between clinical cohorts, healthy individuals may approach the research interview with more formality than do individuals at CHR or with ROP, and thus they adopt a pattern of speaking that employs more Old French words. Individuals at CHR or with ROP would also likely have more experience with clinician-patient interviews, which would not have the same requirements of formality as do other interviews, especially for those in the patient role.

Consistent with this difference of ability is the association of increased Germanic, and reduced Old French, word use specifically with reduced role (but not social) functioning among individuals at CHR and with ROP, associations not seen for lexical diversity or perplexity. Speech etymology differences may reflect a more general difference in professionalism of communication that underlies differences in role functioning. Whereas having a formal vs. informal mode of speech may not be essential to successful social engagement, it may be more important in academic and/or work environments.

### Limitations and future directions

There are several limitations in this study, mostly related to unavailable data. IQ data was available only for participants in Melbourne and Toronto, making up 66% of all participants. Planned collection of cognitive data using the (MATRICS) Consensus Cognitive Battery (MCCB) [31] was impacted by the COVID-19 pandemic, such that data were available only for a subset of the cohort and was not always concurrent with language data. Some of the unexplained variance in speech etymology content might be explained by variance in cognitive domains such as working memory and processing speed, which could respectively influence the tracking of words already used and the retrieval of more uncommon or formal words, both of which could lead to increased Old French word use. These limitations with respect to IQ and cognition are in part mitigated by the absence of findings of association between IQ and etymological variables, and the lack of change in group differences when IQ was adjusted for in models. Additionally, detailed antipsychotic dosage data was not available for participants with ROP which may have revealed a correlation between dosage and etymology content. Further, although all participants were fluent in English, data regarding participants’ native language, language spoken at home, and migration history were not available. These factors might have impacted vocabulary knowledge and the degree of association of speaking English with formality. To address these limitations, future studies could include a cognitive battery, antipsychotic dosages, and more detailed questions about language acquisition.

Future studies could also assess the specificity of these findings of decreased formality among individuals with psychotic symptoms, and its association with role functioning, as these patterns may also be characteristic of other help-seeking individuals who have different symptoms (depression, anxiety) but who likewise have familiarity with clinical interviews. Also, the current study is circumscribed to English-speaking individuals, and it is as yet unclear whether there are equivalent etymological comparisons to be drawn in other languages. It may be that the history of the English language makes it uniquely positioned to index speech formality through etymology, whereas in other languages etymology may provide a different conversational cue. Wiktionary.com contains 8 languages with over 1 000 000 etymology entries each, and an additional 38 languages with over 100 000 entries each. These databases of word etymologies could reveal unique and meaningful patterns of etymology content in other languages.

The Accelerating Medicines Partnership: Schizophrenia (AMP SCZ) consortium [32] may present a unique opportunity for evaluating etymology across languages in CHR individuals, as it entails the collection of recorded open-ended interviews using prompts and approaches adapted from these studies, in seven of Wiktionary’s eight most well-annotated languages (all but Danish). This would allow for testing in a new cohort for increased Germanic and Old French word use in English among CHR individuals, as well as analyses in other languages using well-curated etymology repositories like Wiktionary.com, and correlates with cognition and functional outcomes.

## Conclusion

This study is the first to examine speech etymology content in a clinical cohort. Our findings indicate that proportions of Germanic and Old French word use vary between individuals on the psychosis spectrum and healthy individuals and that this variance is partly attributable to differences in lexical diversity and speech perplexity. Beyond lexical diversity, though, speech etymology content has the potential to operationalize speech formality and may be a factor in reduced role-functioning among individuals with psychosis and at risk. Understanding the relationship of speech etymology content and formality may lead to a better understanding of why role functioning is reduced in psychosis and how these deficits could be treated.

## Supplementary information

Supplemental Materials and Methods

## Data Availability

Data from NIMH-R01-MH107558 and NIMH-R01-MH115332 are being made available through the NIMH Data Archive.
