# Correction: “Ask” or “Inquire”: operationalizing speech formality in psychosis and its risk states using etymology

**DOI:** 10.1038/s44277-025-00027-y

**Published:** 2025-03-19

**Authors:** Matthew Cotter, Alessia McGowan, Zarina Bilgrami, Cansu Sarac, Johanna Bayer, Jessica Spark, Marija Krcmar, Melanie Formica, Kate Gwyther, Jessica Hartmann, Sophia Shuster, Alexandria Selloni, Jai Shah, Shaynna N. Herrera, Patrick McGorry, Alison R. Yung, Barnaby Nelson, Romina Mizrahi, Guillermo Cecchi, Stephen Heisig, Agrima Srivastava, Cheryl M. Corcoran

**Affiliations:** 1 Department of Psychiatry, Icahn School of Medicine at Mount Sinai, New York, NY USA; 2 Department of Psychology, Emory University, Atlanta, GA USA; 3 School of Health Professions, Long Island University, New York, NY USA; 4 Donders Institute for Brain, Cognition and Behavior, Radboud University, Nijmegen, Netherlands; 5 Orygen, The National Centre of Excellence in Youth Mental Health, University of Melbourne, Parkville, VIC Australia; 6 Centre for Youth Mental Health, University of Melbourne, Parkville, VIC Australia; 7 Department of Public Mental Health, Medical Faculty Mannheim, Central Institute of Mental Health, Heidelberg University, Heidelberg, Germany; 8 Department of Psychiatry, McGill University, Montreal, QC Canada; 9 School of Medicine, Deakin University, Waurn Ponds, VIC Australia; 10 Computational Psychiatry and Neuroimaging, IBM, Yorktown Heights, NY USA

**Keywords:** Human behaviour, Data processing

Correction to: *NPP—Digital Psychiatry and Neuroscience* (2024) **2**:17; 10.1038/s44277-024-00018-5 published online 18 October 2024

The funding information was partially missing from this article and should have read “This work was supported by NIMH-R01-MH107558, NIMH-R01-MH115332, and NIMH-R01-MH132239, all to CMC.”

The original article has been corrected.

