# Supplementary material for: “Ask” or “Inquire”: operationalizing speech formality in psychosis and its risk states using etymology
Source: NPP Digit Psychiatry Neurosci. 2024 Oct 18;2:17. doi: 10.1038/s44277-024-00018-5 (PMC11922748; doi:10.1038/s44277-024-00018-5)
Supplement: Supplementary file 1 — Supplemental Materials and Methods [file 44277_2024_18_MOESM1_ESM.docx]

Supplemental Materials and Methods

**Analysis of potential covariates with lexical variables**

Detailed below are methods and results for comparisons of lexical variables (Germanic and Old French word use, lexical diversity, and perplexity) with potential covariates (sex, age, recruitment site, education, racial identity, antipsychotic use, maternal education), along with methods for adjusting lexical variables when significant associations were found.

*Sex*

Across the whole dataset, male participants used more words of Old French origin than did female participants, but male and female participants did not differ significantly in use of Germanic origin words (Table S1). Males and females also did not differ significantly in lexical diversity or perplexity. Since a significant group difference in Old French word use was found, both Old French and Germanic word use were adjusted in male participants by the difference between male and female healthy individuals’ (HC) median value for that variable. Perplexity was also adjusted for sex differences, however lexical diversity was not, as adjusting in this way resulted in an overcorrection due to the large difference between HC female mean and median lexical diversity scores.

*Age*

At the level of the whole dataset, after adjusting for group differences by sex, we found that age was significantly associated with all lexical variables (Table S1). Older individuals demonstrated greater use of Old French words, less use of Germanic words, greater lexical diversity, and greater perplexity. All lexical variables were subsequently adjusted for correlations with age by training a linear regression model on HC data and updating scores in all cohorts using the residuals predicted by the model.

*Recruitment Site*

When comparing group differences in lexical variables between recruitment sites at the level of the whole dataset (after adjusting for sex and age), we found that there were group differences in all variables (Table S1). Participants from Melbourne drove group differences in etymology content, using proportionately more Germanic origin and less Old French origin words than the other two sites. Participants from Toronto exhibited greater lexical diversity and perplexity than the other two sites. To adjust for these differences, we first calculated the difference of median scores between HC participants from Melbourne and HC participants from the other two recruitment sites for each lexical variable. We then adjusted scores in New York and Toronto participants from all cohorts by these differences such that HC participant cohorts were aligned between the sites on each measure.

*Education Duration*

Among participants for whom education data was available (166 HC, 141 clinical high risk (CHR), 88 recent onset psychosis (ROP)), we found at the whole dataset level that etymology content and lexical diversity, but not perplexity, were associated with education duration, after adjusting for associations with sex, age, and recruitment site (Table S1). Those with longer duration of education used more Old French origin words, fewer Germanic origin words, and demonstrated greater lexical diversity. All lexical variables were therefore adjusted for education duration by training a linear regression model on HC data and updating scores in all cohorts using the residuals predicted by the model.

*Racial Identity*

After adjusting for differences in lexical variables by sex, age, recruitment site, and education, we next assessed group differences by racial identity in the subset where available (164 HC, 134 CHR, 86 ROP). Groups differed in proportions of Germanic and Old French word use, as well as lexical diversity, but not perplexity (Table S1). Individuals identifying as Asian used fewer Germanic origin and more Old French origin words than the other groups. White-identifying individuals also used fewer Germanic origin words than those identifying as Black or “Other/more than one race” (OR), and they used more Old French origin words than OR-identifying individuals. Individuals identifying as Black and OR did not significantly differ in proportions of Germanic or Old French word use.

Asian-identifying individuals demonstrated greater lexical diversity than OR-identifying individuals, but not those identifying as Black or White. White individuals had greater lexical diversity than Black individuals and those identifying as OR, and Black and OR-identifying individuals did not differ significantly in lexical diversity.

Since individuals identifying as Black and OR did not differ on any lexical variables, and since these cohorts were small, they were combined before adjusting for group differences by race. To perform the adjustment, we calculated the difference of median scores between HC participants identifying as Asian and HC participants from the other two race categories (White and the combined Black/OR) for each lexical variable. We then adjusted scores in participants identifying as White, Black, and OR from all cohorts by these differences such that HC participant cohorts were aligned between the groups on each measure.

*Maternal Education (As Proxy for Socioeconomic Status)*

After adjusting for differences in lexical variables by sex, age, recruitment site, education duration, and racial identity, we next assessed correlations with maternal education duration in the subset where available (159 HC, 126 CHR, 77 ROP). We found no association with Germanic origin or Old French origin word use at the whole dataset level (Table S1). Greater maternal education was associated with greater lexical diversity, but there was no association with perplexity. Since there were no significant associations with etymology content of speech, no adjustments by maternal education were made.

*Antipsychotic Use*

After adjusting for differences by sex, age, recruitment site, personal education, and racial identity, we assessed group differences by antipsychotic medication prescription in each lexical variable, separately within the CHR and ROP cohorts. In both cohorts, we found no difference in speech etymology content or perplexity by antipsychotic use, however participants who were prescribed antipsychotic medications demonstrated less lexical diversity than those who were not prescribed antipsychotic medications. Since there were no group differences in Germanic or Old French word use, no lexical variables were adjusted for differences by antipsychotic medication prescription.

*IQ Scores*

After adjusting for associations with sex, age, recruitment site, personal education, and race, we assessed correlations with IQ scores in the subset of participants where scores were available (127 HC, 82 CHR, 57 ROP). We found at the whole dataset level that higher IQ scores were associated with greater use of Old French origin words, less use of Germanic origin words, greater lexical diversity, and greater perplexity. As such, we adjusted all lexical variables by training a linear regression model on HC data and adjusting scores in all cohorts using residuals.

**Analyses repeated within recruitment sites**

*Group differences in etymology content and lexical diversity*

As there were significant group differences in Germanic word use, Old French word use, and lexical diversity at the level of the whole dataset, analyses were repeated within recruitment sites. Findings with regard to etymology content in the Toronto and Melbourne datasets were identical to those from the whole dataset – significant group differences (*p* < 0.001), with significant HC x CHR and HC x ROP pairwise differences (both *p* < 0.01), but no CHR x ROP differences. HC participants demonstrated greater Old French word use, reduced Germanic word use, and greater lexical diversity compared to the two clinical cohorts. In the New York dataset, group differences in Old French word use were significant (*p* < 0.05), and there was a trend toward group differences in Germanic word use (*p* = 0.054). Pairwise comparisons of clinical cohorts revealed that those at CHR used proportionately more Germanic (*p* < 0.05) and fewer Old French (*p* < 0.01) words than HC, and those with ROP did not differ significantly from either group.

When examining differences in lexical diversity (as measured with Honoré’s statistic), significant group differences were only identified in the Melbourne dataset (*p* < 0.05), where those with ROP demonstrated less lexical diversity than HC (*p* < 0.05).

*Correlations with role functioning*

In the whole dataset, we identified significant Spearman correlations of Germanic and Old French word use with role functioning within a combined CHR and ROP cohort. We therefore repeated the analyses within recruitment sites. Significant correlations of Germanic and Old French word use with role functioning were identified in New York (*p* < 0.05, *p* < 0.01 respectively) and Melbourne (*p* < 0.05, *p* < 0.01 respectively). No significant correlations were identified in Toronto, which had the smallest n of the three sites, but the direction of correlation was the same as the other datasets, and the effect size of the Old French x role functioning correlation (rho = 0.22) was comparable to that of Melbourne (rho = 0.23) and New York (rho = 0.31). The effect size of the correlation of Germanic word use with role functioning, however, was smaller in Toronto (rho = -0.06) compared to Melbourne (rho = -0.20) and New York (rho = -0.28).

**Supplemental Tables**

*Table S1 – Tests of association between lexical variables and potential covariates*

|  | Proportion Germanic words | Proportion Old French words | Lexical diversity | Perplexity |
| --- | --- | --- | --- | --- |
| Sex |  |  |  |  |
| t score | 1.8 | **-2.4*** | -0.5 | -1.6 |
| Age |  |  |  |  |
| Spearman’s rho | **-0.32***** | **0.35***** | **0.14**** | **0.13*** |
| Recruitment site |  |  |  |  |
| ANOVA F | **14.5***** | **11.6***** | **4.7**** | **5.7**** |
| MB/NY t score | **3.4***** | **-3.1**** | -0.37 | 0.8 |
| MB/TR t score | **5.2***** | **-4.6***** | **-2.9**** | **-2.7**** |
| NY/TR t score | 1.7 | -1.5 | **-2.2*** | **-2.9**** |
| Education |  |  |  |  |
| Spearman’s rho | **-0.22***** | **0.26***** | **0.20***** | **0.05** |
| Racial identity |  |  |  |  |
| ANOVA F | **7.0***** | **8.5***** | **6.2***** | 1.1 |
| Asian/Black t score | **-3.8***** | **3.1**** | 1.7 | - |
| Asian/White t score | **-2.4*** | **3.0**** | -1.7 | - |
| Asian/OR t score | **-3.6***** | **4.7***** | **2.6*** | - |
| White/Other t score | **-2.3*** | **2.9**** | **3.5***** | - |
| Black/White t score | **2.3*** | -1.1 | **-2.8**** | - |
| Black/Other t score | -0.33 | 1.8 | 0.9 | - |
| Maternal education |  |  |  |  |
| Spearman’s rho | -0.08 | 0.04 | **0.12*** | 0.02 |
| Antipsychotic use |  |  |  |  |
| t score | 0.9 | -1.6 | **-2.2*** | 1.0 |
| IQ score |  |  |  |  |
| Spearman’s rho | **-0.28***** | **0.22***** | **0.16**** | **0.14**** |

Note: bold = significant difference or association, with * indicating p < 0.05, ** indicating p < 0.01, and *** indicating p < 0.001. Abbreviations: MB = Melbourne, TR = Toronto, NY = New York, OR = Other/more than one race.

|  | **CHR Symptom Severity** | | | | **ROP Symptom Severity** | | **Global Functioning in combined CHR/ROP cohort** | |
| --- | --- | --- | --- | --- | --- | --- | --- | --- |
| Clinical measure | SIPS Total P | SIPS Total N | CAARMS Total P | CAARMS Total N | PANSS Total P | PANSS Total N | GFS - Role | GFS - Social |
| Germanic Word Use | -0.01 | -0.00 | 0.06 | 0.16 | 0.12 | 0.17 | **-0.19*** | -0.05 |
| Old French Word Use | 0.07 | -0.18 | 0.03 | -0.20 | -0.04 | -0.31 | **0.26**** | 0.12 |
| Honoré's Statistic | - | - | - | - | - | - | 0.14 | - |
| Perplexity | - | - | - | - | - | - | -0.14 | - |

*Table S2 - Spearman correlations between lexical variables and clinical measures*

Note: bold = significant difference or association, with * indicating p < 0.05, ** indicating p < 0.01, and *** indicating p < 0.001
